# Supplementary material for: Silent Tears of Midwives: ‘I Want Every Mother Who Gives Birth to Have Her Baby Alive’—A Narrative Inquiry of Midwives Experiences of Very Early Neonatal Death from Tanzania
Source: Children (Basel). 2023 Apr 10;10(4):705. doi: 10.3390/children10040705 (PMC10137186; doi:10.3390/children10040705)
Supplement: Supplementary file 1 [file children-10-00705-s001.zip › children-2294656-supplementary.pdf]

Supplementary Materials

Table S1. Consolidated criteria for reporting qualitative studies (COREQ): 32-item checklist.

No	Item	Guide questions/description	Answer	Line number in Manuscript
		**Personal Characteristics**		
1.	Interviewer/facilitator	Which author/s conducted the interview or focus group?	JB<br>CB – assisted with audio and discussion after first interviews, together we adapted questions	165
2.	Credentials	What were the researcher's credentials? *E.g. PhD, MD*	JB – Senior Clinical Midwife, CRN, RM BNurs; MAvn Mgt Worked 7 years in this setting<br>CB - Clinical Midwife, RN, Graduate Certificate in Clinical Education, Medical Student - Worked 7 years in this setting	
3.	Occupation	What was their occupation at the time of the study?	JB - Clinical Midwife, RN<br>CB - Clinical Midwife, RN, Medical Student	
4.	Gender	Was the researcher male or female?	Female	
5.	Experience and training	What experience or training did the researcher have?	JB -Master (Research Component)<br>USC Research core course – Clinical Midwife, MAvn Mgt (Research Component)<br>Peer Reviewed Published Journals	

No	Item	Guide questions/description	Answer	Line number in Manuscript
		**Relationship with participants**		
6.	Relationship established	Was a relationship established prior to study commencement?	Yes	137
7.	Participant knowledge of the interviewer	What did the participants know about the researcher? e.*g. personal goals, reasons for doing the research*	Yes, Researcher had worked in this study setting for over 7 years	123
8.	Interviewer characteristics	What characteristics were reported about the interviewer/facilitator? e.g. *Bias, assumptions, reasons and interests in the research topic*	Researcher Bias<br>Interpreter – Kiswahili/English<br>Reflexivity<br>Rigor<br>Research to save babies and meet UN SDG 3	120
		**Domain 2: study design**		
		**Theoretical framework**		
9.	Methodological orientation and Theory	What methodological orientation was stated to underpin the study? *e.g. grounded theory, discourse analysis, ethnography, phenomenology, content analysis*	Narrative Inquiry	110
		**Participant selection**		
10.	Sampling	How were participants selected? *e.g. purposive, convenience, consecutive, snowball*	Purposive	121

No	Item	Guide questions/description	Answer	Line number in Manuscript
11.	Method of approach	How were participants approached? e.g. *face-to-face, telephone, mail, email*	Face to Face, Posters, Email, WhatsApp	123
12.	Sample size	How many participants were in the study?	21	136
13.	Non-participation	How many people refused to participate or dropped out? Reasons?	Nil	N/A
**Setting**				
14.	Setting of data collection	Where was the data collected? e.g. *home, clinic, workplace*	At training clinic at work	115
15.	Presence of non-participants	Was anyone else present besides the participants and researchers?	No	N/A
16.	Description of sample	What are the important characteristics of the sample? *e.g. demographic data, date*	Demographics, skill as midwifes	136
**Data collection**				
17.	Interview guide	Were questions, prompts, guides provided by the authors? Was it pilot tested?	Interview Guide	149
18.	Repeat interviews	Were repeat interviews carried out? If yes, how many?	Yes	200
19.	Audio/visual recording	Did the research use audio or visual recording to collect the data?	Audio device	112

No	Item	Guide questions/description	Answer	Line number in Manuscript
20.	Field notes	Were field notes made during and/or after the interview or focus group?	Yes, before during and afterwards	154
21.	Duration	What was the duration of the interviews or focus group?	Duration 1st interview 40-60 minutes<br>Second interview 20-45 minutes	201-202
22.	Data saturation	Was data saturation discussed?	Yes	157
23.	Transcripts returned	Were transcripts returned to participants for comment and/or correction?	Yes, data was sent via email for participant checking	153
**Domain 3: analysis and findings**				
**Data analysis**				
24.	Number of data coders	How many data coders coded the data?	3<br>JB, CB, JM	160-175
25.	Description of the coding tree	Did authors provide a description of the coding tree?	Themes were labelled	169-175
26.	Derivation of themes	Were themes identified in advance or derived from the data?	Themes derived from data	203-209
27.	Software	What software, if applicable, was used to manage the data?	Excel and word	N/A
28.	Participant checking	Did participants provide feedback on the findings?	Yes	153

No	Item	Guide questions/description	Answer	Line number in Manuscript
		**Reporting**		
29.	Quotations presented	Were participant quotations presented to illustrate the themes / findings? Was each quotation identified? e.*g. participant number*	Yes, quotes used to illustrate each theme<br>Each participant was clearly labelled in the direct quotes	215-286
30.	Data and findings consistent	Was there consistency between the data presented and the findings?	Yes	197-298
31.	Clarity of major themes	Were major themes clearly presented in the findings?	Yes	299-412
32	Clarity of minor themes	Is there a description of diverse cases or discussion of minor themes?	No	N/A
